# Supplementary material for: Are carbon emissions trading and green financial instruments synergistic? -Comprehensive quantitative research based on content analysis
Source: PLoS One. 2024 Mar 7;19(3):e0298601. doi: 10.1371/journal.pone.0298601 (PMC10919601; doi:10.1371/journal.pone.0298601)
Supplement: S1 Data — (ZIP) [file pone.0298601.s001.zip › Data/地级市Z_F工作报告.zip!/╕ú╜¿/╚2├≈╩╨/└┤╫╘╣1⁄2╓┌║┼í╛┬φ┐╦╩2╛▌═°í┐_2005.docx]

各位代表：

　 现在，我代表三明市人民政府向大会报告政府工作，请予审议，并请市政协各位委员和其他列席人员提出意见。

　 一、奋力攻坚、求实进取的重要一年

　 过去的一年，我市各级政府在省委、省政府和市委的坚强领导下，以邓小平理论和“三个代表”重要思想为指导，全面贯彻党的十六大和十六届三中、四中全会精神，树立和落实科学发展观，主动融入海峡西岸经济区建设，迎难而上，顽强拼搏，经过全市人民共同努力，完成或超额完成市十届人大二次会议确定的目标任务，初步统计，全年生产总值376.58亿元，增长10.6%，增幅为1998年以来最高；全社会固定资产投资129.48亿元，增长28.8%；地方级财政一般预算收入17.38亿元，可比增长17.1%；外贸出口2.49亿美元，增长72%，增幅为改革开放以来最高；实际利用外资9116万美元，增长25.1%，增幅为1996年以来最高；社会消费品零售总额117.17亿元，增长9.5%；城镇居民人均可支配收入9968元，增长7.6%；农民人均纯收入3886元，增长9.3%；居民消费价格指数上升4.2%；城镇登记失业率为5.7%；人口自然增长率为5.8‰。一些重要领域、重点项目取得重大突破：

　 ——综合实力进入全国地级以上城市前百位。根据国家统计局发布的消息，我市综合实力居全国地级以上城市第100位。

　 ——三明至福州高速公路全线建成通车。标志着三明的交通条件明显改善。

　 ——向莆铁路前期工作取得重大突破。江西向塘经我市至莆田铁路列入铁道部与省政府签署的福建铁路建设会议纪要，将于“十一五”期间动工建设。

　 ——改造并硬化农村公路1588.6公里，居全省第二位。市级财政筹资7900万元，用于补助农村公路建设。

　 ——三明人民拥有自己的本科大学。三明学院正式成立。

　 ——我市成功获得第一个世界级品牌。经联合国教科文组织评审，泰宁国家地质公园正式进入世界地质公园行列。

　 ——我市被确定为全国集体林区林业产权制度改革唯一试点。全市97.2%的林改村基本完成改革主体任务。

　 ——一批生产性大项目相继投产、动工、签约。明光新型材料公司投产，大亚木业年产45万立方米刨花板项目动工，三化年新增3万吨三聚氰胺项目签约。

　 过去一年，我们突出抓了以下工作：

　 （一）在落实宏观调控中优化经济结构。我们把握宏观调控政策，推动经济结构调整，第二产业比重上升1.2个百分点，私营企业工业产值占规模以上工业产值的比重上升2.4个百分点。落实粮食生产扶持政策，粮食呈现恢复性增长，农民售粮收入增长16.1%。加快培育特色产业，烟叶质量、效益都是历史上最好的一年，肉牛、肉羊出栏分别增长39.5%和23.8%，竹业产值增长28.4%，人工培育红豆杉深度加工产值增长41.3%，莲业产值增长149%。三和食品有限公司、明溪南山农业示范园区，分别成为我市第一家国家级农业产业化龙头企业和第一个国家级农业现代化示范园区。推进老工业基地改造与发展，突出培育冶金及压延业、机械与汽车零配件制造业、林产工业三个产业集群，改造提升纺织、建材、化工三个传统产业，加快发展生物工程、绿色食品加工、新型电子器材三个新兴产业，全市规模以上工业总产值可比增长19.8%，增幅为1998年以来最高。三大产业集群实现产值占规模以上工业总产值的53.9%，比上年提高2.6个百分点，冶金及压延业产值首次突破100亿元。工业经济效益综合指数为145.8%，实现利税29.54亿元。积极发展商贸流通和旅游等服务业，人均消费品零售总额居全省第四位，境内外游客量和旅游总收入分别增长22.1%、18.1%。

　 （二）在克服困难中保持经济有序运行。面对年初高致病性禽流感在周边省份蔓延，我们制定应急预案，严防严控，没有出现高致病性禽流感疫情。面对持续干旱，科学调度人畜饮水和生产用水，适时适地组织人工增雨，开展水利建设年活动，加快“六千”水利工程建设，使旱情得到缓解。面对生产要素紧张，争取建设用地指标，获批农用地转用指标2.61万亩；缓解资金紧缺，区内外金融机构本外币贷款实际增长21.2%；解决电力趋紧问题，规模以上工业用电量增长13.5%，企业万元产值电耗下降13.5%，经济运行没有出现大起大落。

　 （三）在实施项目带动中扩大投资需求。我们跳出三明看三明，立足三明发展三明，全力推动更多项目进入国家和省里盘子。加快突破交通瓶颈制约，交通基础设施投资完成32.09亿元，居全省第一位，邵武至三明高速公路开始路面铺设，泉州至三明高速公路获批动工，永安至武平、永安至宁化高速公路和永安至长汀铁路前期工作取得进展，国省道“改善工程”、交战公路建设等完成投资8605万元。抓好电力开发、电网建设，6座在建大中型水电站总装机容量达46.2万千瓦，街面电站大坝顺利截流，电网改造投资2.4亿元。引导项目向工业集中区集聚，实行大项目全过程服务，15个重点生产性项目建成投产，省里确定的28个工业新增长点项目新增产值占全市新增产值的55%，70个投资千万元以上技改项目全面实施。继续开展项目竞赛活动，全市抓项目、增后劲、促发展的氛围更加浓厚。

　 （四）在推进改革开放中增强发展活力。我们加快产权多元化改革，推进重点企业资产重组，列入年度计划的35家市属工贸企业全部启动改革，三明机床、双轮化机、华橡自控等企业实现优化重组，东闽汽车等劣势企业从一般竞争性领域退出。6家市属国有外经贸企业改革基本完成。水利工程管理体制改革步伐加快，粮食流通体制改革继续深化，粮食安全预警体系进一步完善。通过项目、政策、服务激活民营经济，民营企业完成投资、实现税收分别增长77.3%、21%，商会经济成为民营经济发展的一大亮点。成功举办三明投资贸易洽谈暨商品展销会系列活动，全年内外资动工项目达到188个。外事侨务、对台事务等工作取得进展。

　 （五）在统筹城乡建设中推进城镇化进程。我们按照市区、沙县一体化和市区、永安城市联盟的中心城市发展设想，着手制定市、县一体化概念性规划。推进市区“南拓北扩西进”，抓好台江和东霞片区、贵溪洋小区、小蕉工业集中区规划，促进房地产投资持续增长，商品房投资和销售面积分别增长36.8%、13.2%。投资6500万元，新建、改造城市道路15.1公里，创下历年之最。梯次推进县城和小城镇建设，县城建设步伐加快，首批5个县域次中心试点建设启动，省定中心镇规划建设和省级村镇住宅小区试点工作进展顺利。
